# Skilled Birth Attendants: Who is Who? A Descriptive Study of Definitions and Roles from Nine Sub Saharan African Countries

**DOI:** 10.1371/journal.pone.0040220

**Published:** 2012-07-10

**Authors:** Adetoro Adegoke, Bettina Utz, Sia E. Msuya, Nynke van den Broek

**Affiliations:** Maternal and Newborn Health Unit, Child and Reproductive Health Group, Liverpool School of Tropical Medicine, Liverpool, United Kingdom; Universidad Peruana Cayetano Heredia, Peru

## Abstract

**Background:**

Availability of a Skilled Birth Attendant (SBA) during childbirth is a key indicator for MDG5 and a strategy for reducing maternal and neonatal mortality in Africa. There is limited information on how SBAs and their functions are defined. The aim of this study was to map the cadres of health providers considered SBAs in Sub Saharan Africa (SSA); to describe which signal functions of Essential Obstetric Care (EmOC) they perform and assess whether they are legislated to perform these functions.

**Methods and Findings:**

Key personnel in the Ministries of Health, teaching institutions, referral, regional and district hospitals completed structured questionnaires in nine SSA countries in 2009–2011. A total of 21 different cadres of health care providers (HCP) were reported to be SBA. Type and number of EmOC signal functions reported to be provided, varied substantially between cadres and countries. Parenteral antibiotics, uterotonic drugs and anticonvulsants were provided by most SBAs. Removal of retained products of conception and assisted vaginal delivery were the least provided signal functions. Except for the cadres of obstetricians, medical doctors and registered nurse-midwives, there was lack of clarity regarding signal functions reported to be performed and whether they were legislated to perform these. This was particularly for manual removal of placenta, removal of retained products and assisted vaginal delivery. In some countries, cadres not considered SBA performed deliveries and provided EmOC signal functions. In other settings, cadres reported to be SBA were able to but not legislated to perform key EmOC signal functions.

**Conclusions:**

Comparison of cadres of HCPs reported to be SBA across countries is difficult because of lack of standardization in names, training, and functions performed. There is a need for countries to develop clear guidelines defining who is a SBA and which EmOC signal functions each cadre of HCP is expected to provide.

## Introduction

Sub-Saharan Africa (SSA) contributes to 57% of the 358, 000 global maternal deaths while having only 17% of the global births [1]. Maternal mortality is thus a major public health problem in many SSA countries. The life time risk of dying during pregnancy, childbirth or in the early post natal period is very high in this setting; 1 in 31 compared to 1 in 4300 in developed regions [2]. In addition, more than a third of the approximately 2.65 million stillbirths and 3.3 million neonatal deaths globally, occur in SSA [3], [4].

Most maternal deaths, stillbirths and neonatal deaths are preventable. Access to Skilled Birth Attendance during childbirth and in the immediate post natal period, and access to Emergency Obstetric Care (EmOC) in case of obstetric complications are considered to be effective interventions to reduce the number of global maternal and newborn deaths [1]–[5].

Skilled Birth Attendants (SBA) working together within an enabling environment constitutes Skilled Birth Attendance [6].

A number of studies have shown a correlation between an increased proportion of births attended by SBA and a reduced maternal mortality ratio [7]–[10]. Much of the work to link the availability of SBA and reduction on maternal morbidity and mortality has been guided by observational studies and historical analysis. A reduction in maternal mortality in Scandinavia to less than 300 per 100,000 births in the 1800 s was associated with the development of midwifery as a profession and universal access to attendance of births by a skilled and competent midwife [9]. More recently a correlation has also been noted in low and middle income countries between an increase in the number of births attended by a skilled birth attendant and a reduction in maternal mortality [8], [10]–[12]. Modelling suggests that a critical threshold of having 40% of births attended by a SBA is required [13].

Emergency Obstetric Care is a package of interventions that includes nine ‘signal functions’ including parenteral administration of an oxytocic, antibiotic and anticonvulsant, manual removal of a retained placenta, removal of retained products of conception, assisted delivery, resuscitation of a baby using a bag and mask, blood transfusion and caesarean section [8]. According to a joint statement by WHO, the International Confederation of Midwives (ICM), and the International Federation of Obstetricians and Gynaecologists (FIGO), a ‘Skilled Birth Attendant’ (SBA) is defined as “an accredited health professional such as a midwife, doctor or nurse who has been educated and trained to proficiency in the skills needed to manage normal (uncomplicated) pregnancies, childbirth and the immediate postnatal period, and in identification, management and referral of complications in women and newborns” [14]. The enabling environment is less well defined but includes equipment, drugs and a referral pathway [6].

The proportion of births attended by a SBA is an important indicator for monitoring progress toward MDG 5, and it is one of the indicators tracked by the Countdown to 2015 Initiative with an internationally agreed target of 90% coverage by 2015 [15]. By 2008, 66% of all births globally were attended by a skilled birth attendant. The proportion was low in sub-Saharan Africa (48%) compared to 65%, 93% and 99% in Asia, Region of Americas and Europe respectively [15], [16].

Most SSA countries have adopted the agreed international definition of a SBA [14], [16]. However, human resource shortages have led many of these countries to develop additional and new cadres of staff often referred to as ‘mid-level’ health care providers [17]–[19]. Such cadres may differ greatly between countries in terms of designation, job description and responsibilities [18].

Currently, in SSA countries a wide variety of health care providers provide care during pregnancy, delivery and the postpartum period. There is however a lack of clarity regarding which of these health care providers can be considered an SBA according to the agreed international definition, which key signal functions of EmOC they provide and if they are legislated to perform these functions.

The primary research questions for this study were; 1) which cadres of health care providers are considered to be SBA, 2) which of the EmOC signal functions is each cadre of staff identified reported to perform, 3) which of the EmOC signal functions is each cadre legislated to perform by the relevant regulatory body and 40 what is the length of training and accreditation requirements for each cadre of staff reported to be a SBA.

## Methods

This was a cross sectional, descriptive study which was carried out between 2009–2011 in nine sub-Saharan African countries; Nigeria, Sierra Leone, Ghana, The Gambia, Kenya, Tanzania, Somaliland, Malawi and Zimbabwe. The countries included in the study were purposively selected to represent West, East and Southern Africa.

A structured self-administered questionnaire, which was in English, was used for data collection. It was sent to Key Informants (KI) in each of the nine countries. (Table 1) A total of 5 questionnaires were sent to each country. KI were purposively selected to include; personnel working for the reproductive and child health (RCH) unit of respective ministries of health, senior staff of teaching institutions (medical and midwifery/nursing schools), health care providers or managers of either the referral, regional or district hospitals, as well as technical staff of development agencies.

**Table 1:** Profile of Key Informants and number of respondents by country.

Country	Profile of Key Informants	Number ofrespondents
**Nigeria**	Director Department of Reproductive Health Federal Ministry of Health; Registrar Nursing andMidwifery Council; Accrediting Officer Nursing and Midwifery Council; Head Association ofObstetrician Gynaecologists; Head Nursing and Midwifery School.	5
**Ghana**	Head Department of Reproductive Health Ghana Health Services; Head Midwifery School; Head CommunityNursing School; Head Medical and Dental Council; Head Association of Obstetricians and Gynaecologists.	3
**Sierra Leone**	Director Department of Reproductive Health Ministry of Health; Reproductive Health ManagerMinistry of Health; Head Midwifery School; National Family Planning CoordinatorMinistry of Health; District Nursing Officer.	3
**Gambia**	Principal Secretary Ministry of Health; Manager Reproductive Health Unit; Head Nurses and Midwives Council;Head Medical Board; Head Community Health Officer Program.	3
**Kenya**	Director Department of Reproductive Health Ministry of Health; Senior Health Advisor Department ofReproductive Health Ministry of Health; Head Association of Obstetricians and Gynaecologists;Head Nursing Council; District Nursing Officer.	3
**Tanzania**	Manager Department of Reproductive Health Ministry of Health; Head Nurses and Midwives Council;Head Nursing School; Head Department of Obstetrics and Gynaecology Medical School; DistrictMedical and Nursing Officer.	5
**Somaliland**	Manager Department of Reproductive Health Ministry of Health; Head Nurses and Midwives Council;Head Nursing School; Head Department of Obstetrics and Gynaecology Medical school; DistrictMedical and Nursing Officer.	4
**Malawi**	Head Department of Reproductive Health Ministry of Health; Head College of Nursing and Midwifery;Head Medical Council; Head, Nurses and Midwives Council; District Nursing Officer.	4
**Zimbabwe**	Director Department of Reproductive Health Ministry of Health; President Association of Obstetriciansand Gynaecologists; Head Department of Obstetrics and Gynaecology Medical School; Head NursesCouncil; Head Medical and Dental Practitioners Council.	4

Information collected included: the definition of a SBA, cadres of staff reported to be SBA in each country, the existence of regulatory bodies, which of the nine EmOC signal functions (Table 2) were expected to be performed by each cadre and whether these cadres were legislated to perform these signal functions. Information on entry requirements and length of training for the various cadres of SBAs was also collected.

**Table 2:** Signal functions for Essential (or Emergency) Obstetric Care.

Basic EmOC Services	Comprehensive EmOC Services
1. Parenteral antibiotics	All included in Basic EmOC (1–7)
2. Parenteral oxytocic drugs	plus:
3. Parenteral anticonvulsants	
4. Manual removal of placenta	8. Caesarean Section
5. Removal of retained products (e.g. by manual vacuum aspiration)	9. Blood Transfusion
6. Assisted vaginal delivery (Vacuum delivery)	
7. Newborn resuscitation using bag and mask	

Source: WHO (2009) Managing emergency obstetric care: a handbook.

The questionnaires were sent both as soft and hard copies to the Key Informants in each country and returned by email or post. Reminders both by phone and email were sent to those who had not returned the questionnaire by December 2010.

When a particular question was answered differently by the key informants in the same country, representatives of the Ministry of Health were contacted to clarify the information. When consensus was not reached even after further probing, all answers were retained and such cases are highlighted in the results.

Data was entered and analysed using SPSS version 18.

Verbal informed consent was obtained from all the participants after explaining the purpose of the study.

## Results

### Response Rate

Out of a total of 45 questionnaires 19 (42.2%) were returned after the first round and a further 15 after a reminder giving a total response rate of 75.6%.

### Background Characteristics of Participating Countries

Maternal mortality ratio was high in all nine countries ranging from 350 per 100,000 live births in Ghana to 1,200 per 100,000 live births in Somaliland (Table 3). The proportion of births reported to be assisted by skilled health personnel varied widely between the countries, ranging from 40% to 69%, with only one country, Zimbabwe, having coverage of more than 60%.

**Table 3:** Background maternal health indicators of nine participating countries.

Country (DHS year)	Total Population(millions)†	TFR	Estimated deliveriesper year	Percent of birthsassisted by skilledhealth personnel	Estimated MMR(per 100,000 live births)*
**West Africa**					
Nigeria (2008)	154.7	5.7	6,281,993	40	840
Ghana (2008)	23.8	4.0	734,188	59	350
Sierra Leone (2008)	5.7	5.1	179,439	43	970
Gambia††		5.9	61,000	57	400
**East Africa**					
Kenya (2008/09)	39.8	4.6	1,385,111	44	530
Tanzania (2010)	43.7	5.4	1,666,458	51	790
Somaliland ‡‡	8.9	6.7	395,00	33	1200
**Southern Africa**					
Malawi (2010)	15.3	5.7	598,326	73	510
Zimbabwe (2005/06)	12.5	3.8	388,206	69	790

All the data in the table unless indicated were compiled from the recent DHS Survey, DHS StatCompliler (www.measuredhs.com).

† Source: The World Bank, 2009 (www.worldbank.org).

* Source: WHO, 2011: World Health Statistics 2011 (www.who.int/whosis/whostat/2011/en/index.html).

†† WHO Country Cooperation Strategy for Gambia, WHO African Office, 2009; MOHSW Gambia; and MICS.

‡‡ Somaliland is not recognised as a state by the United Nations; hence, the above statistics apply to the recognised Somali Republic (Somalia and Somaliland). Source of Data is from the WHO Making Pregnancy Safer Country Report.

### Cadres of Health Care Provider Considered to be SBA

The range of cadres of health care providers who were reported to be “Skilled Birth Attendants” (SBA) differed greatly among the countries (Table 4). Apart from Nigeria which had only four cadres of health providers who were considered SBA, the rest of the countries had between 6 and 11 different cadres of health care providers, reported to be SBA In three settings there was lack of consensus around some cadres: 1) in Sierra Leone some KI reported that MCHA were considered to be SBA while other KI said they were not; 2) in Tanzania respondents from the district and teaching institutions reported that there is no cadre called ‘registered midwife’ and that this would be either a ‘registered nurse midwife’ or ‘enrolled nurse midwife’. However, KI at the Ministry of Health level reported the cadre ‘registered midwife’ existed and 3) in Kenya some KI reported there was a cadre called ‘enrolled nurse midwives’ while others did not.

**Table 4:** Cadre of staff considered a skilled birth attendant present at each country and total number of years of training.

	Nigeria		Ghana		Sierra Leone		Gambia		Kenya		Tanzania		Somaliland		Malawi		Zimbabwe	
	Cadre	Total years of training	Cadre	Total years of training	Cadre	Total years of training	Cadre	Total years of training	Cadre	Total years of training	Cadre	Total years of training	Cadre	Total years of training	Cadre	Total years of training	Cadre	Total years of training
Obstetrician	**√**	**10**	√	9	**√**	**9–10**	√	10	**√**	**9–10**	√	9–10	**√**	**Differ** 5	√	9–10	**√**	**9–10**
General Doctors	**√**	**7**	√	6.5	**√**	**6**	√	7	**√**	**6**	√	6	**√**	**7**	√	6	**√**	**6**
Assistant Medical Officers (AMO)	–		–		–		–		–		√	5	–		–		–	
Clinical Officers/Health officers	–		–		**√**	**3**	–		**√**	**3**	√	3	**√**	**4.5**	√	4	**√** †	**6**
Medical Assistant	–		√	3–4	–		–		–		–		–		√	2	–	
Assistant Clinical Officers/Clinicalassistants	–		–		–		–		–		√	2	–		–		–	
Enrolled midwife	–		–		–		√	3	**√** †	**2**	–		–		√†	2	–	
Enrolled nurse	–		–		–		√	2	**√** †	**2**	√†	1–2	–		√† ^2^	2	–	
Enrolled nurse midwife	–		√† **^1^**	4	**√**	**4.5**	√	3	√*		√	4	–		√† ^3^	3	–	
Registered midwife	**√**	**3**	√	3	**√**	**4.5**	√	4.5	–		√*		–		–		–	
Registered general nurse	–				–		√	3	**√** ††	**3.5**	–		**√** †	**3**	–		**√**	**3**
Registered nurse midwife	**√**	**4**	√	3	**√**	**4.5**	√	4.5	**√** † **^1^**	**4–5**	√^4^	4–5	**√**	**4**	√	4–5	**√**	**4**
Registered community health nurse	–		–		–		–		**√** 1	**4**	–		–		–		–	
Nurse midwife technicians	–		–		–		–		–		–		**√**	**2**	√^3^	3	–	
Nurse technicians	–		–		–		–		–		–		–		√^2^	2	–	
Community midwives	–		–		–		–		**√** *†	**2–3**	–		**√**	**1.5**	–		–	
Community health nurse midwife	–		√^1^	4	–		√	3	–		–		–		–		–	
Auxiliary midwife	–		√	2	–		–		–		–		–		–		–	
Mother & Child Health Aide (MCHA)	–		–		**√** *	**2**	–		–		√†	1	–		√† ^6^	1	–	
State enrolled community nurse	–		–		**√**	**2.5**	–		–		–		–		–		–	
State certified nurse midwives	–		–		–		–		–		–		–		–		**√** †	**3.5**

† This cadre was previously trained but the training has stopped, and the cadre is still in the system.

†† The government was reported to have stopped training this cadre of health care provider, but training continues at some private and/or faith based hospitals.

* No consensus was reached between Key Informants of the study regarding the existence of this cadre or if it is regarded as SBA.

*† Different cadres of retired nurses called to serve in the community.

1 Training curriculum has changed with a larger community health component, so the cadre which is graduating from the same institutions is now referred to as a Kenya Registered Community Health Nurse (KRCHN) or Ghana Community Health Nurse Midwife (CHNM) instead of as previously - a Registered Nurse Midwife.

2 Enrolled nurse (EN) training curriculum has been changed to Nurse Technicians (NT) &.

3 Enrolled Nurse Midwife (ENM) training curriculum has been changed to Nurse Midwife Technicians (NMT). The same institutions which were training EN and ENM are now training NT &NMT.

4 Training duration has changed from four to three years from the 2010 intake of RNM diploma students.

5 Include medical doctors with long experience in Obstetrics and Gynaecology (OBGYN), certificate in OBGYN after medical training and specialist training in OBGYN.

6 Training was done for only one intake and then stopped.

### Training

The length of training was 9–10 years for obstetrician gynaeclogists and 6–7 years for medical doctors, but varied substantially for the other cadres of health care providers even when these had the same name (Table 4). Training for a registered diploma nurse midwife for example was three years in Ghana, four and a half years in Sierra Leone and Gambia and four years in the other countries. Except for Nigeria, in all other countries included in the study, nurse-midwives undergo training in general nursing first and then have an additional midwifery training period ranging from 12–18 months (Table 4). The total number of years of professional training for the cadre called “clinical officer” also differed between countries; three years in Tanzania and Kenya, four years in Malawi, four and half in Somaliland and six years in Zimbabwe. In Somaliland and Zimbabwe a health care provider needs to be a trained nurse or nurse-midwife with field experience before being allowed to commence clinical officer training while in Tanzania, Kenya and Malawi a candidate could apply directly after secondary school. Different lengths of training were also observed for medical assistants, enrolled nurse midwives, and community midwives (Table 4).

In a number of countries additional new cadres of health care providers were emerging. In Ghana for example, training for registered nurse midwives (RNM) has been scaled down and a new cadre of providers (trained by the same institutions) are trained: the Community Health Nurse Midwife. In Kenya, the RNM training was stopped, and the new cadre of providers currently trained are called Kenya Registered Community Health Nurses, while in Malawi training of Enrolled Nurse Midwives has changed and the new cadre is called a Nurse Midwife Technician. Tanzania on the other hand, has not changed the name of the cadre RNM, but it has shortened the length of training from 4 to 3 years.

Training of some cadres which are considered SBAs has completely stopped in some countries, with previously trained cadres continuing to exist in the system but not being replaced. Clinical Officers in Zimbabwe, registered general nurses in Somaliland, enrolled nurses and enrolled midwives in Kenya, Tanzania and Malawi respectively, are examples of SBAs for which training has stopped, (Table 4). In Kenya, the government has stopped training general nurses, however church and other private colleges continue to train this cadre of SBA.

### Signal Functions: Performance and Legislation

Tables 5 & 6 depict the signal functions reported to be performed by different cadres of clinicians. In all the nine countries, obstetricians are legislated to and reported to perform all nine signal functions. Medical Doctors are also legislated to perform all nine signal functions with the exception of Zimbabwe where they are not legislated to perform vacuum extraction until they have obtained additional training. In some countries, despite being legislated to do so, medical doctors do not in practice perform all the signal functions, especially signal function number 4 (manual removal of placenta), 5 (removal of retained products) and 6 (assisted vaginal delivery). Signal functions performed by clinical officers or health officers varied among countries; in Malawi and Zimbabwe they can perform a caesarean section while in Sierra Leone, Tanzania and Kenya they are not allowed to do so.

**Table 5:** Signal functions reported to be performed by different cadres of clinicians (Western Africa).

	Nigeria										Ghana										Sierra Leone										Gambia									
	D	1	2	3	4	5	6	7	8	9	D	1	2	3	4	5	6	7	8	9	D	1	2	3	4	5	6	7	8	9	D	1	2	3	4	5	6	7	8	9
Medical Doctors	√	√	√	√	√	√	√	√	√	√	√	√	√	√	√	√	√	√	√	√	√	√	√	√	○	√	○	√	√	√	√	√	√	√	√	√	√	√	√	√
Assistant Medical Doctors																																								
Clinical or Health Officers																					○	√	√	√	X	X	X	√	X	√										
Medical Assistants											√	√	√	√	√	√	√	√	X	√																				
Assistant Clinical Officers																																								
Enrolled midwife																															√	√	X	X	X	X	X	√	X	X
Enrolled nurse																															√	√	X	X	X	X	X	√	X	X
Enrolled nurse midwife											√	√	√	√	√	√	√	√	X	√	√	√	√	√	√	X	X	√	X	X	√	√	√	√	√	X	X	√	x	√
Registered midwife	√	√	√	√	√	○	X	√	X	√	√	√	√	√	√	√	√	√	X	√	√	√	√	√	√	#	#	√	X	√	√	√	√	√	√	√	√	√	X	√
Registered general nurse																															√	√	X	X	X	X	X	√	X	√
Registered nurse midwife	√	√	√	√	○	○	X	√	X	√	√	√	√	√	√	√	√	√	X	√	√	√	√	√	√	#	#	√	X	√	√	√	√	√	√	√	√	√	X	√
Registered community health nurse																																								
Nurse midwife technicians																																								
Nurse technicians																																								
Community midwives																																								
Community health nurse midwife											√	√	√	√	√	√	√	√	X	√											√	√	X	X	X	X	X	√	X	√
Auxiliary midwife											√	√	√	X	X	X	X	X	X	X																				
Mother & Child Health Aide (MCHA)																					√	√	√	√	X	X	X	√	X	X										
State enrolled community nurse																					√	√	√	√	X	X	X	X	X	X										
State certified nurse midwives																																								
CHEW	#	#	#	#	#	X	X	X	X	X																														
JCHEW	#	#	#	#	#	X	X	X	X	X																														
Medical attendant																																								

Key: **√**: Performing and legislated; **○:** Not performed but legislated #: Performing but not legislated; x: Not performed and not legislated *: Only those who had attended training in BEMOC or LSS or MVA can perform these signal functions.

D = Deliveries, 1 = Parental antibiotics; 2 = Parental oxytocic drugs; 3 = Parental anticonvulsants; 4 = Manual removal of retained placenta; 5 = MVA; 6 = Assisted vaginal delivery (vacuum extraction); 7 = Newborn resuscitation; 8 = Caesarean section; 9 = Blood transfusion.

* No legislative body so the tick stands for training received & perform function; **x** Training was not received and not performing; **√:** Performing but not trained; **x** Few performed after LSS training.

**Table 6:** Signal functions reported to be performed by different cadres of clinicians (East, Central and Southern Africa).

	Kenya										Tanzania										Malawi										Zimbabwe										Somaliland*									
	D	1	2	3	4	5	6	7	8	9	D	1	2	3	4	5	6	7	8	9	D	1	2	3	4	5	6	7	8	9	D	1	2	3	4	5	6	7	8	9	D	1	2	3	4	5	6	7	8	9
Medical Doctors	√	√	√	√	√	√	√	√	√	√	√	√	√	√	√	√	○	√	√	√	√	√	√	√	√	√	√	√	√	√	√	√	√	√	○	○	X	√	√	√	√	√	√	√	√	√	√	√	○	√
Assistant Medical Doctors											√	√	√	√	√	√	○	√	√	√																														
Clinical or Health Officers	√	√	√	√	○	√	X	√	X	√	√	√	√	√	√	√	X	√	X	√	√	√	√	√	√	√	√	√	√	√	√	√	√	√	○	√	X	√	√	√	√	√	√	√	○	X	X	√	X	√
Medical Assistants																					√	√	√	√	√	√	√	√	X	√																				
Assistant Clinical Officers											√	√	√	√	√	X	X	√	X	X																														
Enrolled midwife	√	√	√	√	*	*	*	√	X	√											√	√	√	√	*	*	*	*	X	√																				
Enrolled nurse	√	√	√	√	*	*	*	√	X	√	√	√	√	√	√	X	X	√	X	√	#	√	○	X	X	X	X	X	X	√																				
Enrolled nurse midwife											√	√	√	√	√	√	○	√	X	√	√	√	√	√	*	*	*	*	X	√																				
Registered midwife											√	√	√	√	√	√	○	√	X	√																														
Registered general nurse	√	√	√	√	*	*	*	√	X	√																					√	√	√	○	X	X	X	√	X	√	√	√	√	√	√	*	*	√	X	√
Registered nurse midwife	√	√	√	√	√	√	√	√	X	√	√	√	√	√	√	√	○	√	X	√	√	√	√	√	√	√	√	√	X	√	√	√	√	√	√	X	X	√	X	√	√	√	√	√	√	*	√	√	X	√
Registered community health nurse	√	√	√	√	√	√	√	√	X	√																																								
Nurse midwife technicians																					√	√	√	√	*	*	*	*	X	√											√	√	√	√	√	X	X	√	X	√
Nurse technicians																					#	√	○	X	X	X	X	X	X	√																				
Community midwives	√	√	√	X	√	X	X	√	X	X																															√	√	√	√	√	X	X	√	X	√
Community health nurse midwife																																																		
Auxiliary midwife																																									#	#	X	X	X	X	X	X	X	X
Mother & Child Health Aide (MCHA)											√	√	√	√	√	X	X	√	X	X	√	√	X	X	X	X	X	X	X	X																				
State enrolled community nurse																																																		
State certified nurse midwives																															√	√	√	X	X	X	X	√	X	√										
CHEW																																																		
JCHEW																																																		
Medical attendant											#	#	#	X	X	X	X	X	X	X																														

Key: **√**: Performing and legislated; **○:** Not performed but legislated #: Performing but not legislated; x: Not performed and not legislated *: Only those who had attended training in BEMOC or LSS or MVA can perform these signal functions.

D = Deliveries, 1 = Parental antibiotics; 2 = Parental oxytocic drugs; 3 = Parental anticonvulsants; 4 = Manual removal of retained placenta; 5 = MVA; 6 = Assisted vaginal delivery (vacuum extraction); 7 = Newborn resuscitation; 8 = Caesarean section; 9 = Blood transfusion.

* No legislative body so the tick stands for training received & perform function; **x** Training was not received and not performing; **√:** Performing but not trained; **x** Few performed after LSS training.

**Figure 1:**
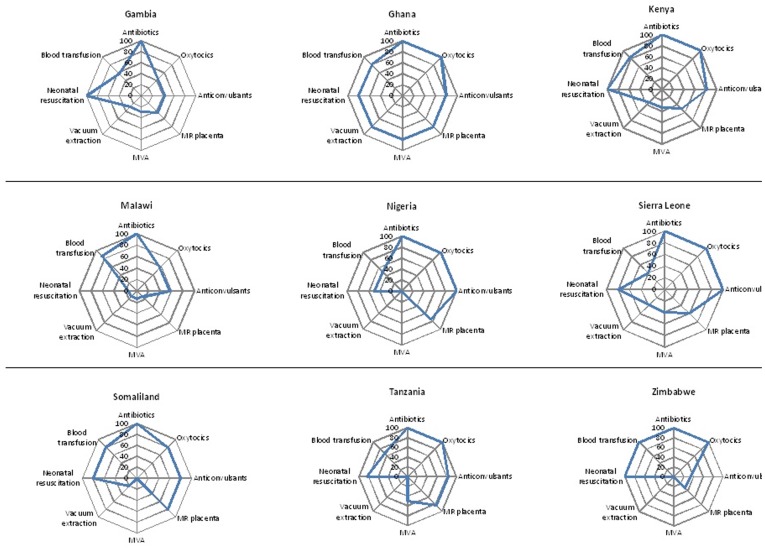
Percentage of different types of cadres of Nurses/Midwives who perform each of the eight signal function by country.

The signal functions performed by different cadres of nurses are shown in Tables 5 & 6 and Figure 1. Nurses are not currently legislated to perform a caesarean section in any of the surveyed countries. The number of EmOC signal functions performed by different cadres of nurses varied within and between countries. Even the cadres of nurses with the same title were observed to provide different numbers of signal functions. RNMs provide five out of eight signal functions in Nigeria and Zimbabwe, seven out of eight in Sierra Leone, Tanzania and Somaliland and all eight in Ghana, Gambia, Kenya and Malawi respectively. In all countries, most nurses or midwives are legislated and are performing signal functions number 1 and 2 (administer parenteral-antibiotics and uterotonic drugs).

Performance of the remaining signal functions among different cadres of nurses varied substantially among countries. The RNMs in Gambia, Ghana, Kenya, Malawi and Tanzania for example were legislated to and did provide signal functions number 4 and 5 (manual removal of placenta and removal of retained products), where as they were legislated to but not perform these functions in Nigeria. The same cadre, i.e. RNM, were not legislated to perform signal function number 6 (assisted vaginal delivery e.g. vacuum extraction) in Nigeria, Gambia and Zimbabwe, legislated to but did not perform signal function 6 in Tanzania and perform it though not legislated to do so in Sierra Leone.

Figure 1 shows the proportion of different cadres of nurse-midwifery health care providers who can provide a signal functions for each country and for each of the eight EmOC signal functions they could potentially perform (i.e. excluding caesarean section). In all the countries, parenteral antibiotics could be offered by all cadres of nurses. However in three of the nine countries provision of an oxytocic is not a signal function currently provided by 100% of the available cadres of nurses expected to function as SBA (20% in Gambia, 60% in Malawi, 80% in Somaliland). Similarly, only 80% of the available cadres of nurses administered anticonvulsants in Ghana, Kenya, Tanzania and Somaliland, and this is only 40% in Zimbabwe and 60% in Malawi respectively. The proportion of available cadres of nurses performing manual removal of placenta, removal of retained products, and assisted vaginal delivery (vacuum extraction) was low in all the countries with the exception of Ghana, ( Figure 1).

Tables 5 & 6 also outline other cadres of nurses such as enrolled midwives, enrolled nurse midwives and nurse midwife technicians who were traditionally not trained to perform signal functions 4, 5 and 6, but who are reported to now provide these signal functions if they have attended additional in-service training either in Life Saving Skills (LSS) or in Basic Emergency Obstetric Care (BEmOC). However, the legislation status has not yet changed. This was observed in Kenya, Malawi and Somaliland.

Except for the cadres: obstetricians, medical doctors and registered nurse- midwives, there was lack of consensus among KI regarding which signal functions were being provided by 0 cadres of staff and which of these they were legislated to perform. This was especially so for signal functions 3 (parenteral anticonvulsants), 4 (manual removal of placenta), 5 (removal of retained products) and 6 (assisted vaginal delivery) respectively.

Finally in some countries it was noted that there were cadres of providers which were not reported to be SBA but who were conducting deliveries and providing some of the EmOC signal functions, especially in rural and hard to reach areas (Tables 5 & 6). Examples include auxiliary midwives and medical attendants in Somaliland and Tanzania who conducted deliveries and Community Health Extension Workers (CHEWs) in Nigeria who conduct deliveries and can administer parenteral antibiotics, oxytocics and anticonvulsants as well as perform manual removal of retained placenta.

### Regulatory Bodies

Except for Somaliland, all countries had regulatory bodies for most cadres of health care providers reported to be SBA, (Table 7). In Tanzania clinical officers, clinical assistants and Maternal and Child Health Aides (MCHA) did not have a regulatory body while in other countries where these cadres exist, a regulatory body was in place. While registration is required to practice for nearly all the cadres and in all countries, the requirements for renewal of registration differed between countries. Continuous educational development points were required for renewal of registration for obstetricians and medical doctors in Nigeria and Zimbabwe, fees were charged in Malawi and there were no specified requirements for renewal in Tanzania and Sierra Leone. For nurses, requirements for renewal of licence also differed between countries. Members were required to renew their registration annually in some countries, while for others this was every three years (Table 7). Evidence of continuous medical education and payment of a fee were the most common requirements for renewal or continuation of registration.

**Table 7:** Regulatory bodies and renewal requirements by cadre and country.

	Nigeria		Ghana		Sierra Leone		Gambia		Kenya		Tanzania		Malawi		Zimbabwe	
	RB*	Requirementfor renewal	RB	Requirementfor renewal	RB	Requirementfor renewal	RB	Requirementfor renewal	RB	Requirementfor renewal	RB	Requirementfor renewal	RB	Requirementfor renewal	RB	Requirementfor renewal
Obstetrician	Y	Yearly, PD points	Y	Yearly, PD points	Y	None	Y	None	Y	Yearly, PD points	Y	None	Y	Yearly, fees	Y	Yearly, PD points
General doctors	Y	Yearly, PD points	Y	Yearly, PD points	Y	None	Y	None	Y	Yearly, PD points	Y	None	Y	Yearly, fees	Y	Yearly, PD points
Assistant medical officers (AMO)											Y	Every 3rd year, fees				
Clinical officers/health officers					Y	None			Y	Yearly, PD points	N	N/A	Y	Yearly, fees	Y	Yearly, fees, PD points
Medical assistant			Y	Every 3rd year, PD points									Y	Yearly, fees		
Clinical assistants											N	N/A				
Enrolled midwife							Y	Yearly, PD points	Y	Every 3rd year, fees, PD points			Y	Yearly, fees, PD points		
Enrolled nurse							Y	Yearly, PD points	Y	Every 3rd year, fees, PD points	Y	Every 3rd year, fees	Y	Yearly, fees, PD points		
Enrolled nurse midwife			Y	Every 3rd year, PD points	Y	Every 3rd year, fees, PD points	Y	Yearly, PD points			Y	Every 3rd year, fees	Y	Yearly, fees, PD points		
Registered midwife	Y	Every 3rd year, fees, PD points	Y	Every 3rd year, PD points	Y	Every 3rd year, fees, PD points	Y	Yearly, PD points			Y	Every 3rd year, fees				
Registered nurse							Y	Yearly, PD points	Y	Every 3rd year, fees, PD points					Y	Yearly, fees, PD points
Registered nurse midwife	Y	Every 3rd year, fees, PD points	Y	Every 3rd year, PD points	Y	Every 3rd year, fees, PD points	Y	Yearly, PD points	Y	Every 3rd year, fees, PD points	Y	Every 3rd year, fees	Y	Yearly, fees, PD points	Y	Yearly, fees, PD points
Registered community health nurse									Y	Every 3rd year, fees, PD points						
Nurse midwife technicians													Y	Yearly, fees, PD points		
Nurse technicians													Y	Yearly, fees, PD points		
Community midwives									Y	Every 3rd year, fees						
Community health nurse midwife			Y	Every 3rd year, PD points												
Auxiliary midwife			Y	Every 3rd year, PD points												
Mother & Child Health Aide (MCHA)					Y	Every 3rd year, fees, PD points					N	N/A	Y	Yearly, fees, PD points		
State enrolled community nurse					Y	Every 3rd year, fees, PD points										
State certified NM															Y	Yearly, fees, PD points

* RB  =  Regulatory body.

## Discussion

The proportion of births attended by a Skilled Birth Attendant (SBA) is a key indicator for measuring progress towards MDG5. In practice a variety of health care providers provide care for women during pregnancy, childbirth and the puerperium. This study has for the first time described the different cadres of staff reported to be SBA in nine countries in sub-Saharan Africa and explores their roles in terms of reported signal functions of Emergency Obstetric Care performed. As many as twenty one different cadres of health providers were reported to be SBA differing substantially with regard to given name, length of training, signal functions being provided or legislated to provide.

To achieve a significant increase in the proportion of births attended by a SBA, countries need to ensure adequate numbers are trained and deployed per population [6], [7], [14]. A greater number of cadres of health care providers considered to be SBA available in a country might be expected to improve SBA coverage, but this study illustrates that this is currently not the case. Zimbabwe with 6 cadres of health care provider reported to function as SBA reports 69% of births attended by SBA, while Kenya, Tanzania and Malawi with 9–11 different cadres report coverage rates of 44%, 51% and 56% respectively [16], [20].

There was no direct correlation between the total number of cadres of health care providers reported to be SBA and availability of EmOC signal functions. With the exception of Ghana, most of the cadres reported to be SBA are not reported to provide the full range of EmOC signal functions [17]. Signal functions such as manual removal of placenta, manual vacuum aspiration (MVA) for retained products of conception in case of an incomplete miscarriage and ability to conduct assisted vaginal delivery (e.g. vacuum extraction) were the least reported to be performed across all countries. Previous research that assessed the availability of EmOC signal functions in various African and Asian countries also noted these were the least available EmOC signal functions in many settings [21], [22]. To improve coverage, it is important to examine if the skills and competency of all (or some of) the cadres of health care providers expected to function as SBA needs to be expanded, as well as the legislative and enabling environment including the availability of equipment and drugs [7], [23].

In some settings cadres reported to be SBA were not effectively utilized to offer life saving procedures including EmOC signal functions because of existing policy. Though trained in and reported to be capable of performing the signal functions there was lack of matching legislation to allow such health care providers to perform EmOC signal functions they were in principle able to perform. This was noted even where health care providers had received additional in-service training in EmOC. This could be seen as a missed opportunity as increased availability of these critical signal functions could be achieved relatively easily with change in legislation and ‘task shifting’ or ‘up-skilling’ of existing cadres of staff [7], [18], [23].

Failure to enable cadres of staff reported to be SBA to provide EmOC signal functions when they are able to do so and when these competencies are needed not only affects the availability of services in health facilities, but also the quality of care and the utilization of services by women. Studies have shown that quality of care i.e. availability of drugs, equipment and skilled and competent providers has a major influence on women’s choice of a delivery facility [24], [25]. In Western Tanzania a study conducted by Kruk *et al* (2009) showed that, concern about poor quality of care caused 41% of the women who delivered at health facilities to bypass the nearest facility which offered only some of the obstetric services and delivered at facilities which were further away [25]. Also a large proportion of those who delivered at home cited quality of care as one of the major deterrence factors.

Another important finding in this study is the fact that in some countries cadres of health care providers who were not reported to be SBA are known to conduct deliveries and provide several of the EmOC signal functions. This seems to be mainly among the disadvantaged poor and rural women where there is a shortage of more highly trained staff [14], [18]. At a minimum, these countries need to clarify what level of care such staff can and are legally able to provide. In addition the opportunity for competency based training must be considered [26].

The creation of new cadres of SBA in addition to existing cadres in the system will further create confusion on signal functions and it adds difficulties in monitoring performance and in supervision [18]. Health authorities need to plan in advance which EmOC signal functions new cadres of health care providers will be expected to perform. This will need to be reflected in the training curricula and the level of competency should be communicated to health leaders at the region, district and at facility level. Researchers should also be aware that this dynamic change of names, job descriptions and functions performed by health care providers, makes it difficult to compare studies and reports on skilled birth attendants and skilled birth attendance within and between countries.

### Implications for Policy and Practice

Although all of the countries included in this study report having agreed and adopted the WHO definition of a SBA [10], we noted that it was a challenge to get consistent information regarding which signal functions are expected to be performed by each identified cadre of staff considered to be a SBA. Staff working for the Ministry of Health sometimes held a view that was different to that of senior health care providers at regional or district level. This signifies a lack of communication between policy makers and implementers. The common reason cited for this was a lack of clear written guidelines and policies regarding the roles and responsibilities of an SBA. There is an urgent need therefore for the Ministries of Health, and health regulatory bodies such as the Medical Council, the Nursing and Midwifery Council and the Professional Associations of obstetrician-gynaecologists to formulate and agree clear guidelines and policy. It is understandable that countries are striving to increase the number of SBA and/or expand the remit of existing cadres of health care providers in order to meet the goal of SBA coverage of 90% by 2015. However in order to ensure effective and good quality clinical care is indeed available, an emphasis on competence and quality of care must be maintained. Many countries are encouraging women to deliver at health facilities implying that the facilities are well equipped and have skilled health personnel. However not all cadres of staff considered to be SBA are able to offer the full range of signal functions of emergency obstetric care needed to prevent maternal and newborn mortality and morbidity and it is important to ensure the right skills mix. Again lack of clear legislation was one of the most important factors identified by KI in this study and this will need to be addressed. If coverage with good quality skilled birth attendance and EmOC is to be realized.

### Limitations of the Study

This study has some limitations and was very much an initial exploration to try and ascertain which cadres of health care providers were reported by Key Informants (KI) at policy and legislation level to be expected to function as a SBA. Information about cadres of staff considered to be SBA and which signal functions they provided relied on reports by these KI and were not verified by observation, interview or skills assessment of health care providers themselves. We may have therefore over or under estimated the number of signal functions that are provided in practice. We also realise that despite a good response rate and broad range of KI, the total number of KI was limited. Future research should combine key informant interviews and facility level observation to give a clearer picture of whether these health care providers really are working as SBA, the presence or lack of an enabling environment and actual performance of the EmOC signal functions. At the outset of this study, we had not expected to find such a wide range of variety with regard to cadres of staff considered to be SBA. Given that there is only a very limited overlap between countries making comparison across the whole of SSA very difficult, for future in depth studies, regional comparisons might be more relevant.

### Conclusions

There are currently more than twenty different cadres of health care providers who are reported to be work as a SBA in the nine surveyed sub-Saharan African countries. Comparison of these cadres across countries is difficult because of different names, roles and responsibilities. There is a distinct lack of clarity regarding which cadre is legislated to perform which signal functions of EmOC highlighting the need for the Ministries of Health, Nursing and Midwifery Councils and Professional Associations to work together to develop and disseminate policy and guidelines. Further studies are needed to document whether cadres of health care provider in principle reported to be SBA are really able to provide skilled birth attendance for which an enabling environment is also needed and which of whether these health care providers have the necessary skills and competency to effectively work as SBA and which of the EmOC signal functions they should and can perform to ensure improved maternal and newborn health outcomes.
